# Co-creation environment with cloud virtual reality and real-time artificial intelligence toward the design of molecular robots

**DOI:** 10.1515/jib-2022-0017

**Published:** 2022-10-04

**Authors:** Akihiko Konagaya, Gregory Gutmann, Yuhui Zhang

**Affiliations:** Molecular Robotics Research Institute, Co., Ltd., 4259-3, Nagatsuta, Midori, Yokohama, Japan; Keisen University, 2-10-1, Minamino, Tama, Tokyo, Japan

**Keywords:** cloud virtual reality, co-creation environment, molecular robotics, real-time artificial intelligence, virtual reality simulation

## Abstract

This paper describes the design philosophy for our cloud-based virtual reality (VR) co-creation environment (CCE) for molecular modeling. Using interactive VR simulation can provide enhanced perspectives in molecular modeling for intuitive live demonstration and experimentation in the CCE. Then the use of the CCE can enhance knowledge creation by bringing people together to share and create ideas or knowledge that may not emerge otherwise. Our prototype CCE discussed here, which was developed to demonstrate our design philosophy, has already enabled multiple members to log in and touch virtual molecules running on a cloud server with no noticeable network latency via real-time artificial intelligence techniques. The CCE plays an essential role in the rational design of molecular robot parts, which consist of bio-molecules such as DNA and protein molecules.

## 1 Introduction

Biomolecular design and representation have been studied in various fields including drug discovery, life, chemical sciences, and noble material development, to name a few [1]. Illustrated models and computer graphics are often used in manuscripts and computer-aided design systems to capture the shapes and functionalities of biomolecule models [2]. However, our intuitive understanding of biomolecules, except for the experts in biochemistry, is often limited due to the lack of a basis in reality, in the sense that one cannot touch the biomolecules as they can with everyday objects in the real world.

Virtual reality (VR) is one of the promising ways to solve the above issues by representing biomolecules as virtual objects, which are visible and touchable with the help of a VR headset with a hand motion recognition device [3]. According to our early experience with touchable biomolecular VR objects, they are very helpful to enhance our intuitive understanding of target biomolecules when aligning a VR object onto a microscope image and reproducing X-ray crystal structures of a protein complex from VR monomer objects, to name a few.

**Figure 1:**
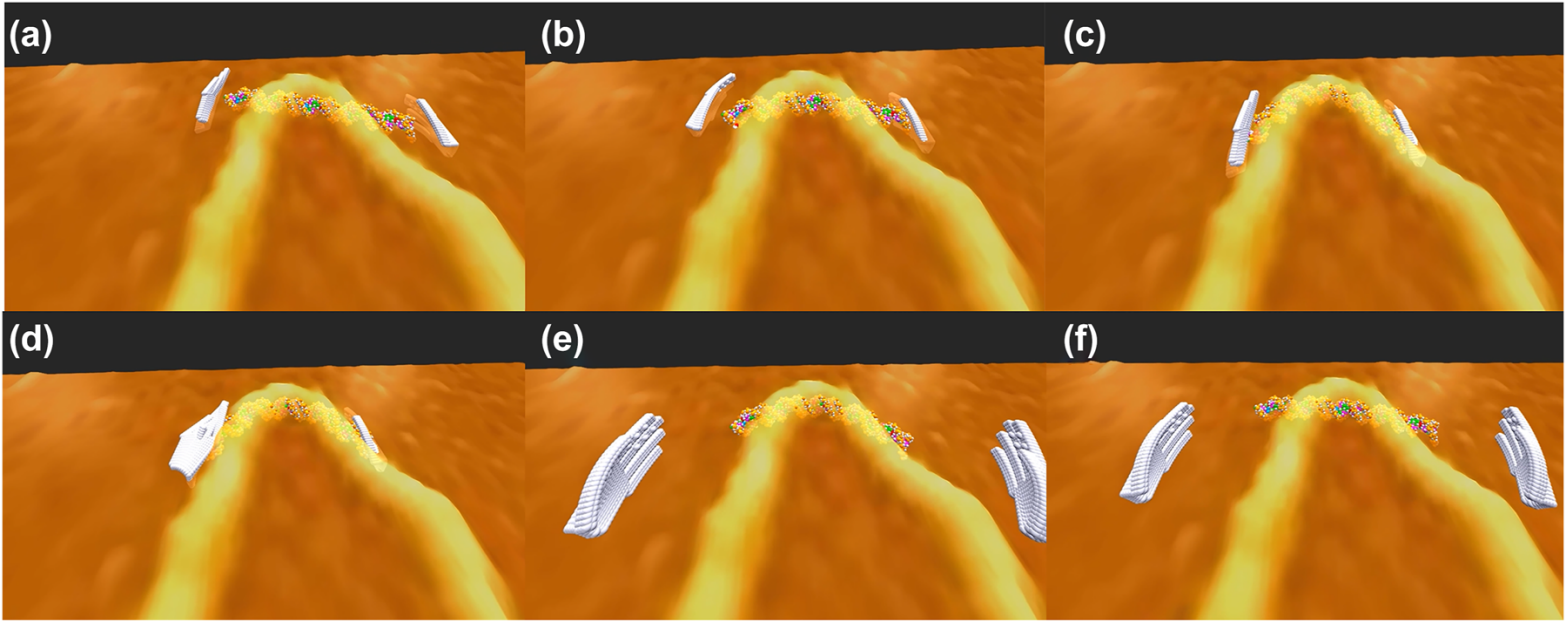
Alignment of a DNA double-strand and an AFM image with virtual hands, bending process (a) and (c), and recovering process (b) and (d): the DNA double-strand returns to its original form when the pressure from the virtual hands is released due to the tensegrity representation [4] of the DNA double-strand in VR simulation.

Figure 1 shows the alignment of a VR DNA fragment and a VR AFM image of a phage DNA double strand. The alignment not only enhances the intuitive interpretation of AFM images but also reminds us of the research questions about the sharp bending of DNA double helix: How much force is required for bending a DNA double-strand in a shape? What causes such sharp bending on a DNA helix? Is there any kink DNA structure at the top of the bending? Is there any sequence dependency on the curvature of DNA helix bending, and so on?

**Figure 2:**
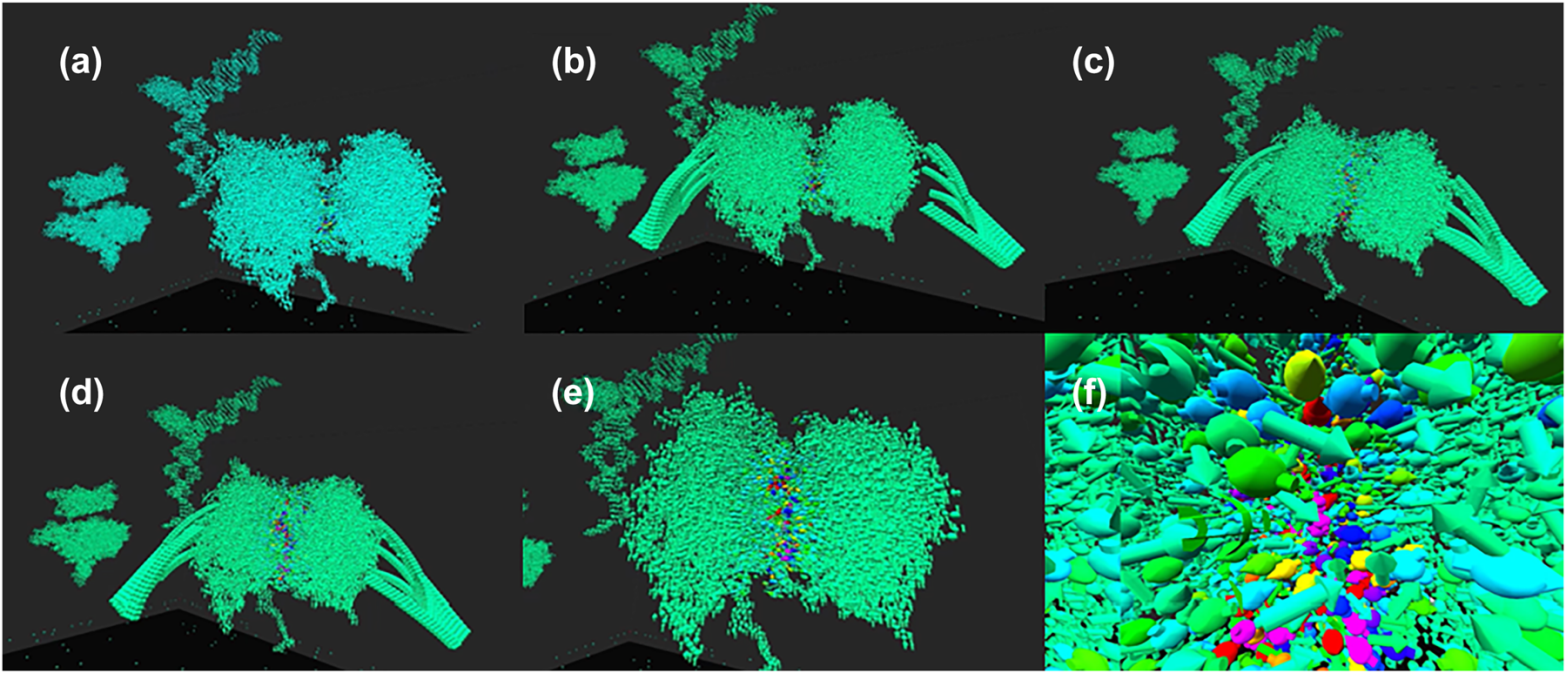
A tubulin-dimer reproduction from alpha and beta tubulins with virtual hands (a–d) and more zoomed-in (e) and (f): colored atoms with arrows indicate interaction force among atoms ranging from most attractive (purple), attractive (blue), neutral (green), repulsive (yellow) to most repulsive (red) according to visible light wavelengths.

Figure 2 shows the occurrence of binding force among alpha-tubulin and beta-tubulin when forming a tubulin dimer with an X-ray structure. It should be noted that the dimer is constructed from two tubulin monomers by adjusting the binding interface with virtual hands. Interestingly, a strong binding force suddenly appears when alpha-tubulin and beta-tubulin bind at the same angle as observed in the X-ray structure. Any misalignment with regard to the rotational angle and distance of the two tubulins results in the binding force, known as the Van der Waals force, suddenly disappearing. It also reminds us about further questions such as what if a GTP (guanosine triphosphate) connecting the tubulin dimer is replaced by a GDP (guanosine diphosphate), how hydrogen bonds play in this binding, and what if other tubulin dimers are aligned alongside the tubulin dimer, to name a few.

**Figure 3:**
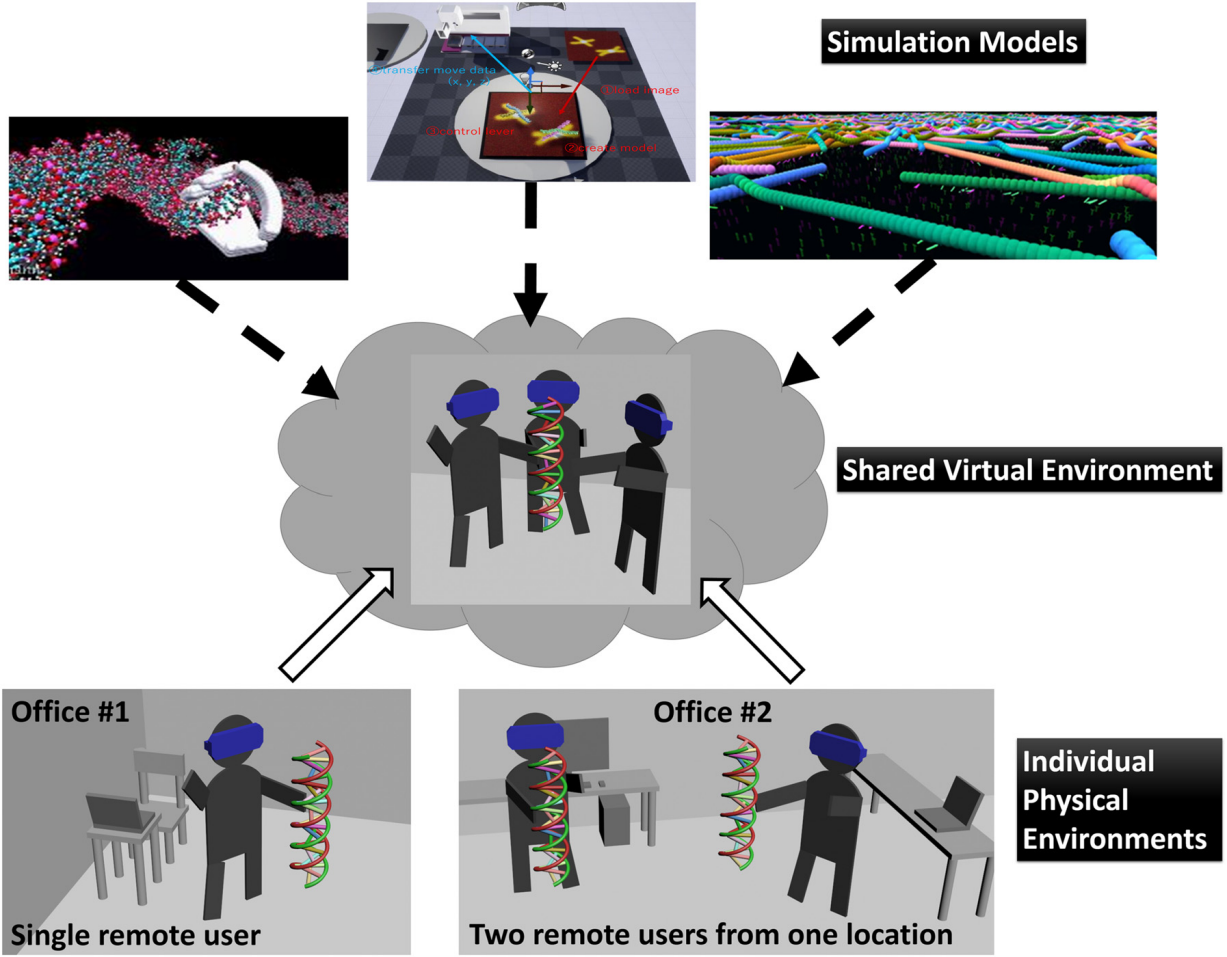
Conceptual design of CCE.

In order to answer the above questions, we have developed a cloud-based VR co-creation environment in which multiple members can log in to the molecular simulation of virtual molecules running on a cloud server as if they are all working together in the same room (Figure 3). The environment enables the members to share and touch the virtual molecules with their virtual hands concurrently.

One of the plausible applications of the CCE is a bio-molecular design system in which one can design useful molecular parts with virtual DNA and proteins in advance of actual wet experiments. Since all molecular interaction results simulated in the CCE are only hypothetical works, careful interpretation and investigation from various backgrounds including biochemistry, biophysics, molecular design, and simulation are needed.

In this sense, the multiple-user environment is mandatory for the CCE.

Due to the breadth of our CCE project, this paper is intended to be a position paper to explain our design philosophy, and it will then be followed by technical and experiment-based papers as each component is tested to evaluate the merit of our design choices. The organization of this paper is as follows. Section 2 presents the design philosophy of the CCE and related works. Section 3 introduces the challenges of simulation accessibility, cloud VR, and the real-time inference and training technology used in our cloud VR. Section 4 discusses the status of our current CCE prototype. Section 5 covers our VR viewer for microscope images. Section 6 gives a brief introduction to molecular robotics which our CCE is being used for. Section 7 provides our conclusions and discusses future work.

## 2 CCE design philosophy

Rational molecular design has already played major roles in countless fields, for example, in medicine and material sciences [5]. However, owing to the growing interest in design at the molecular scale and the complexity of molecular parts, such as those in micromachines and molecular robots, it is important to improve our tools to enable the creation of molecular designs that are more complex and layered than in past work [3]. To address this key issue of developing tools for rational molecular design, our proposed platform, a cloud VR CCE augmented by real-time AI, offers enhanced perspectives, knowledge creation, and guided experimentation.

### 2.1 Enhanced perspectives

**Figure 4:**
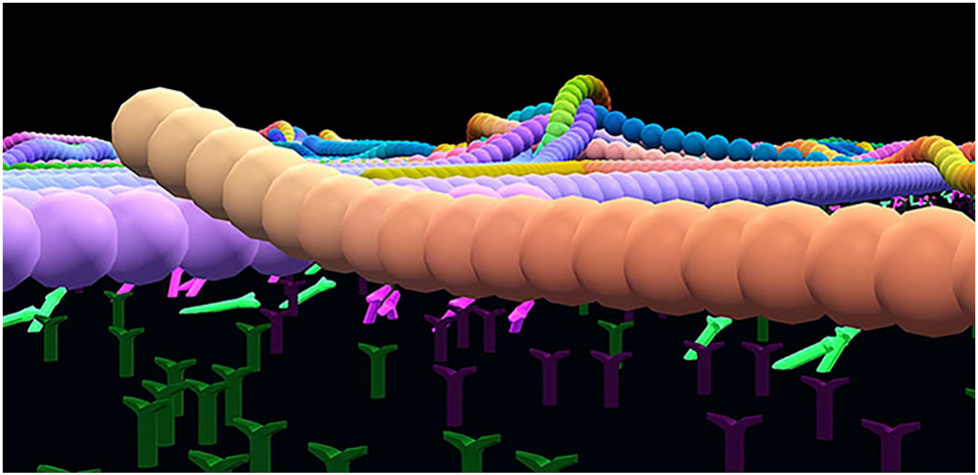
An up-close view of motor proteins pushing microtubules in VR; each sphere represents a portion of a microtubule about 140 nm in length, and the Y-shaped objects represent immobilized kinesins propelling microtubules on a glass surface [[3], 62 supplemental materials].

Enhanced perspectives can be provided by using VR in combination with an interactive simulation system [3, 6]. In VR, a researcher can move freely through the molecular world by walking, turning, and moving their head to see any perspective in a natural way. For example, microtubule-microtubule interactions in mobility assays are difficult to visualize clearly from microscope images and video data. However, VR allows us to show the microtubule-microtubule interactions propelled by motor proteins right in front of an observer (Figure 4). This type of insight can help clarify dynamics that are otherwise not visible or easily measurable because many hypotheses can be tested and viewed at microscopic and macroscopic scales. VR also encourages enhanced perspectives because molecular research can be conducted in a multi-sensory environment that includes visual indicators and haptics [7, 8]. Recent research in psychology and neuroscience has shown that our attention is improved when we engage in multi-sensory processing [9, 10].

### 2.2 Knowledge creation

Knowledge creation is one of our objectives for developing the cloud VR CCE. The creation of knowledge by connecting users virtually has been studied previously using grid computing, especially for drug discovery and design [11]. Similar to how the grid connects many people for collaboration, our own multiple-user CCE using cloud computing can do the same. Our CCE aims to provide a modern grid platform with VR for knowledge creation based on previous work in this area [12, 13].

A cloud-based VR CCE can bring people together to share ideas or knowledge in a way that may not be or seem possible in everyday life. To give an example with respect to our own work, when most think about “molecular sciences” it may conjure an image of a group of experts in lab coats speaking in jargon and writing down complex chemical structures, like what is seen in movie laboratories. We believe that if molecular science appeared more approachable, it would allow for more people to participate and understand what is happening at the cutting edge of research.

The game-like interactive nature of our co-creation platform makes it a candidate for encouraging wider participation, and it may help to simplify multi-disciplinary collaboration and allow more people of all ages to enter into or learn about molecular science [14]. Although this is unlikely to increase the number of molecular scientists suddenly, the research community would benefit if gamification were to generate more interest in molecular sciences in the general population [15, 16]. Cloud VR co-creation spaces can do this for nearly any topic because anything that can be imagined can be realized in a virtual space.

### 2.3 Guided experimentation

Guided experimentation can also be important in accelerating and communicating findings [17, 18]. Guided experimentation is the combination of human-in-the-loop simulation for guiding dynamics and the communication of findings to guide others in their own work. AI could also be used as a guide for interpreting changes in the system and providing new perspectives for further testing [19], and this is an area for future exploration.

We have developed the CCE platform including human-in-the-loop VR simulation, cloud-based VR implementation, and real-time artificial intelligence-controlled hand motion prediction from a scratch in order to deal with the molecular interaction among millions of atoms in VR. In our own prototype, guided experimentation is already essential in the parameter searches and tuning of coarse-grained molecular dynamics simulations. Because the spatial and temporal scales of coarse-grained simulations can vary widely, people often need to find new models and parameters that can reproduce natural phenomena, and initially, there is often no set definition to tune simulations automatically with algorithmic or AI methods [20].

The cloud aspect of our work touches on many areas of study, such as cloud-based collaboration, VR, and remote operation. These technologies have a wide range of applications, and thus there are many groups working to provide solutions with one or more of these technologies. Cloud-based VR could be used for connecting people across society and sharing information with greater immersion [21]. One meta-analysis examined computer-supported collaborative learning and found that it improved learning [11]. A second area could be overcoming social issues and constraints, allowing virtual interactions, such as medical appointments, consultations, and learning [22]. A third possible benefit is that it could aid in the analysis of big data by allowing the visualization and interaction with data in three dimensions [23].

These benefits could expand the possibilities open to people if they could share VR objects and touch them naturally without feeling a disconnect due to network latency. The following sections focus on these points from the viewpoint of VR molecular modeling and real-time inference and training for remote virtual hand operation.

### 2.4 Related works

Table 1 summarizes the characteristics of our own interactive molecular simulation and other published or commercial tools for muti-user facilities, VR facilities, interactive simulation, and building molecules live.

**Table 1:** High-level overview of several interactive molecular systems. “Large-scale” indicates scales on the order of hundreds of thousands or more atoms or particles.

Work	Multi-user	VR	Interactive	Building	Comments
			simulation	molecules live	
Our work	Local and remote	O	O	X	Large-scale variable coarse-graining at VR rates
Deeks et al. [24, 25]	X	O	O	X	Docking, two molecules
O’Connor et al. [6] Bennie et al. [26]	Local	O	O	X	Tens to thousands of atoms, established models
Schroeder [27]	X	X	O	X	1–2500 non-bonded atoms
Luehr et al. [28]	X	X	O	X	Small (dozens of atoms)
Dreher et al. [29]	X	X	O	X	Large-scale but limited rates
Stone et al. [30]	X	X	O	X	Multiple molecules
OVITO [31]	X	X	O	X	Particle-based systems at various scales, including large scales
Samson connect [32]	X	O	O	O	Various scales, including large scales
Nanome [33]	Local and remote	O	X	O	Design & visualization
Kut’ák et al. [34]	X	O	X	O	Design & visualization
UnityMol [35–37]	Local and remote	O	X	O	Design & visualization

Of the systems in Table 2, only three others than ours have multi-user support [6, 33, 36]. However, for our own team’s needs, these other tools are insufficient. The system reported by O’Connor et al. [6] only has local multi-user support and their simulation scales are drastically different from our own, although it appears that any models can be incorporated. Nanome [33] does not support large scales and interactive simulation which is an important feature for our team’s experimentalist. The objective of UnityMol [35–37] was very similar to ours but their approach was completely different in the sense that we don’t use any game engine in order to deal with million number of virtual objects and their interaction in real-time.

**Table 2:** Molecular robot research.

Achievement	Method	Impact	Team
Amoeba-like robot that can change shape	DNA-modified motor proteins and microtubules, light-activated DNA clutch	First amoeba-like molecular robot assembled with biomolecules	Tohoku University team [38]
Spindle-shaped liposome robot that can extend filopodia-like protrusions	Light-activated actin filament	Large cell-shape deformation and fast movement without using motor proteins	Nagoya University team [39]
Self-propelled giant liposome	Light-activated peptide nanofiber growth	Actin comet-like movement with peptide aggregates	Tottori University team [40]
Muscle-like actuator	Genetically modified motor protein filaments and microtubules	Strong enough to change the shape of micromachines at the millimeter scale	JAIST and Osaka Universities team [41]
Star-like microtubule structures	DNA modified microtubules and DNA nanostructures powered by ATP	Actuator with muscle-like contraction	Hokkaido and Kansai Universities team [42]
Molecular robot swarm	Boundary conditions and photosensitive DNA linkers	Large-scale molecular motion control	Hokkaido and Kansai University team [43, 44]

Some of the other systems use VR [6, 24, 32–34] and others use interactive simulation [6, 24, 32]. Nanome [33] and the system reported by Kut’tak et al. [34] do not include interactive simulation and primarily focus on design instead; the molecular structures are built in VR, and then these results are used for simulations in other software, such as conventional molecular dynamics simulations or coarse-grained simulations, such as OxDNA.

## 3 Molecular modelling on cloud VR

Owing to the recent boom in cloud-based services, smaller and more cost-effective cloud solutions are appearing [45, 46], bringing the benefits of the cloud to more people. For us, this means that we can offload the computational work from the client to the cloud so that the clients do not need powerful hardware. This also enables the creation of shared environments for bringing people together because the cloud server functions as a mutual connection point. To exploit these advantages for molecular modeling of robot parts, the problems with molecular modeling, scalability, and network performance must be solved.

The scalability of the molecular simulation system is critical when designing molecular parts for molecular robots. Conventional molecular dynamic simulation targets from X-ray crystallography have molecular weights from tens to thousands of Daltons [47]. In contrast, molecular parts in our simulation, such as DNA origami, often contain more than half a million atoms [48]. More than a million particles are needed to reproduce non-trivial motion patterns similar to those observed in experimental data such as microtubule motility assay, even if we use coarse-grained simulations [20]. Previously, this would have required multiple high-performance servers; however, the rapid advancement in general-purpose graphics processing units has enabled us to execute large-scale molecular dynamics simulations with visualizations on a high-end gaming PC or laptop [3]. Our cloud-based VR service is our next step in making our system available to even more people. The simulation is offloaded to the cloud where it can be shared by multiple users that only need a VR-capable device with network connectivity.

Consequently, network performance is critical in cloud VR system development because poor network performance leads directly to a poor user experience [49]. Bandwidth and latency are two of the main factors in network performance, although there are others, such as packet loss.

**Figure 5:**
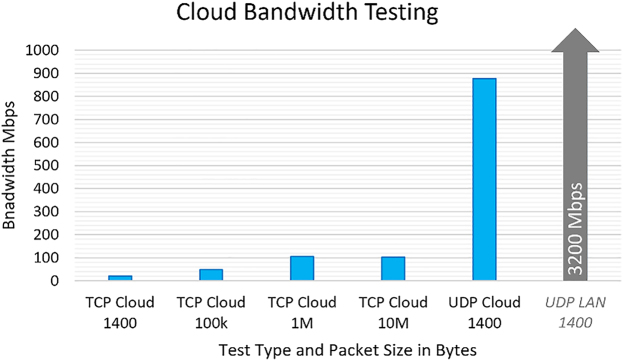
Comparison of TCP and UDP network performance. The bandwidth tests were conducted between a server in Ishikari, Japan, configured with a bandwidth of 1.5 Gbps, and a client in Kanagawa, Japan, with a 1 Gbps internet connection.

Network bandwidth affects how much data can be sent to the client and how frequently the data can be updated. Therefore, our bandwidth needs are partly tied to the simulation scale, but, in general, if there is more bandwidth available, the user has a smoother experience. There is a limit to the effect of the amount of available bandwidth because updating faster than the simulation rate or the refresh rate of the user’s screen/VR goggles is wasteful. An early evaluation of the VR cloud bandwidth performance is shown in Figure 5. The actual bandwidth that is available in the cloud can be completely different from that in local area networks (LANs) because the bandwidth is sometimes limited by network protocols or for other reasons, even if higher network bandwidth hardware is configured.

Latency can create a time discrepancy between user actions and the presentation of the simulation results. If the latency is high, the user is more susceptible to suffering from VR sickness and the overall experience is poorer [50]. Thus, network latency must be minimized. To achieve this, we are proposing the use of an artificial neural network for predicting user motion to negate the effects of latency.

### 3.1 Real-time inference and training for remote virtual hand operation

To minimize network latency, we used our real-time inference and training to predict remote virtual hand motions [51]. As we discussed in previous work, our platform’s prediction requirements are different from those in most other research in this area [51].

In order to touch a virtual object with a virtual hand in the VR simulation running on a remote server, the virtual hand must reach the virtual object before the user’s hand reaches the position of the virtual object to overcome the network latency issue. By having the future state (prediction) of the user, the cloud server begins to create an updated simulation state ahead of time so that even after the time required to create a new simulation state and send it to the user has passed, it is still up to date.

We believe that this is a crucial technology that will help us to realize our CCE; the key to a CCE’s success is the user experience. If the interaction in the environment is not fluid like a real-life experience, users will likely leave the platform prior to even testing interactions or the simulation models that have been implemented. Consequently, this was our first priority in developing our CCE and the other components are now actively in development.

In our past work on prediction [51], we presented quantitative results, but because the platform as a whole was being developed at that time, qualitative results, such as user testing, were beyond the scope of the work. The recent progress on our CCE will allow us to test it with a real-world platform with many users and evaluate our machine learning prediction work more extensively.

## 4 CCE prototype

This section provides a complete overview of our current CCE prototype, which is a custom implementation that has been built by our group using languages and application programming interfaces, such as C++ and CUDA, for the simulation, and DirectX 12 for the user VR rendering.

### 4.1 Hardware resources and requirements

**Figure 6:**
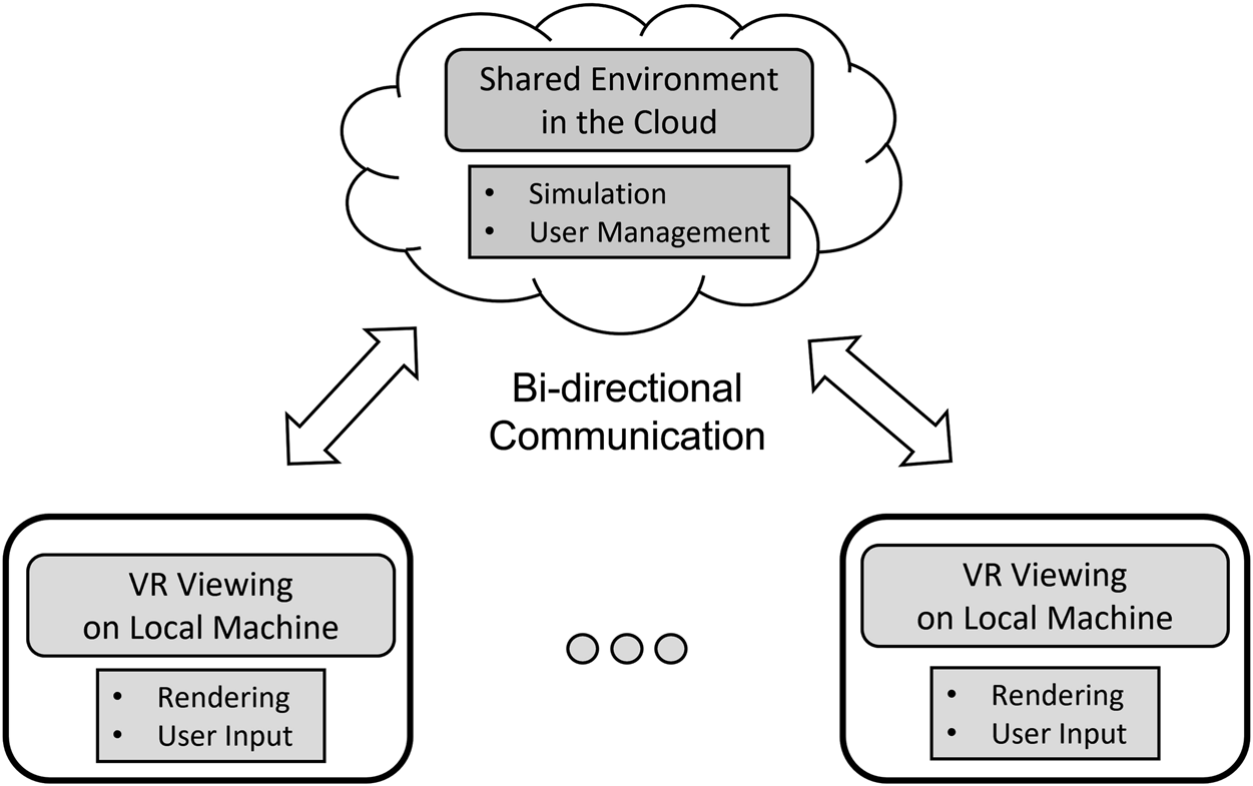
Overview of our current CCE system configuration.

Our CCE hardware consists of a central server, which in our case is in the cloud; however, it could also be a local server depending on users’ resources and security concerns. As for the users who connect to the cloud, their machines are responsible for rendering the simulation environment reported by the server. A visual overview with additional information is shown in Figure 6.

### 4.2 Technical design considerations

**Figure 7:**
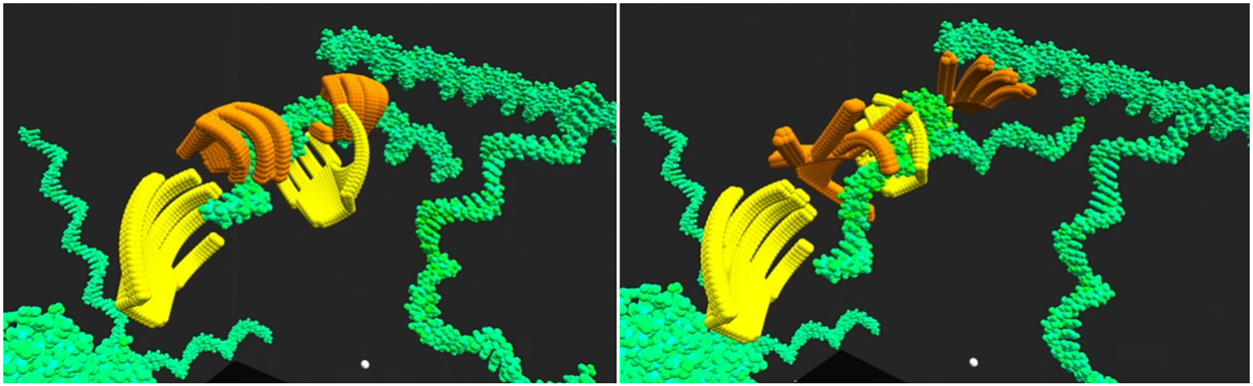
Snapshots from a video of two users passing a DNA strand in our VR CCE prototype. At the time of publishing, the video can be found at https://youtu.be/C3UCthg7aEA. A maintained list of links can be found at https://en.molecular-robot.com/en/news/.

In contrast to some game streaming services, such as GeForce Now or Google Stadia, and other visualization works [52, 53], we chose to use local rendering for several reasons. First, the purpose of remote rendering is to offload computationally intense work from the client; however, our rendering is lightweight because it has been built from scratch for our use. Second, the desired resolution of VR is high, and up to 8 K is commercially available. This would pose another challenge on its own because the rendered frames of a particle simulation contain high frame-to-frame entropy, and thus it is difficult to reach good compression ratios without introducing noticeable noise, as seen by the differences in the quality of our live VR and recordings uploaded to YouTube (See Figure 7); a more detailed examination of compression rates and quality is needed though in the future. Lastly, minimizing the latency tied to the user’s head motion down to single-digit milliseconds is critical for a good VR experience. With our local rendering, this is not a problem, only the simulated particles could be affected by latency, which has a smaller impact on the user experience. As network performance increases, remote rendering for VR cloud environments [54] might become more feasible, then we may reconsider using remote rendering for our CCE use cases.

**Figure 8:**
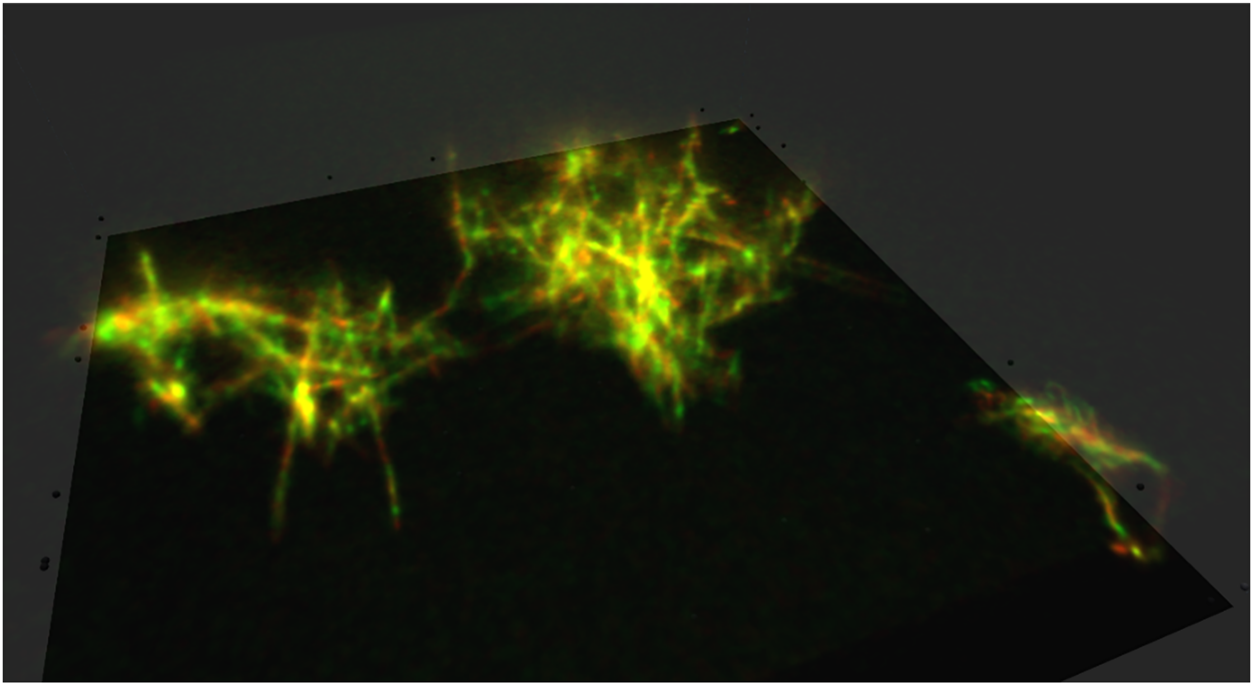
A view of a star-like microtubule rendered in our VR confocal image viewer mode, courtesy of Dr. Yutaka Ueno (AIST) [43].

Some modern multiplayer games include dynamic environments that are simulated based on physical interactions; however, the degree to which the environment can be changed is still limited compared with reality [55]. In addition, in these games, the changes at a high level can be seen by all users, but the fine details, such as particle systems, are often unique representations generated locally on each user’s machine. In our own system, everything is dynamic, but the representation could be regarded as simpler because everything is represented by particles. Another key difference is that in our CCE, every user sees the same dynamics within the environment. This is important for research communication, as it allows users to demonstrate dynamics in the environment to other users and discuss them interactively.

### 4.3 User interaction and experience

Currently, users can load VR molecular objects in Protein Data Bank format [56], and then run the simulation system to view and interact with the contents of the file. The current model includes the Lennard–Jones potential, Coulomb force, and bonded terms. In the future, we are aiming to allow the user to choose from a range of simulation models and simulation matter to load into the CCE, for example, atoms, molecules, microtubules, DNA, user-defined coarse-grained objects, and others. In terms of user interaction, users can currently physically walk around or use a controller to move within the VR environment. They can use their hands to directly interact with the simulation matter using a Leap Motion device for hand tracking, from which we generate a kind of hand-shaped molecule for each hand. Snapshots from a video are shown in Figure 7.

For our prototype in its current state, the primary hypothesized benefit is the ability to share dynamic VR objects easily*.* This allows us to probe systems live and interactively in any way that we choose to observe the resulting dynamics. The shared dynamic VR objects can be handled through the conventional wide area network or the Internet. This is one of the major differences between our approach and the game streaming or remote rendering approaches [52, 53, 55], which would require a high-performance network such as a wired LAN or local 5G network if they were to deal with high-resolution VR images with millions of moving particles.

### 4.4 Ongoing and future work

The most important next step for our CCE is to conduct user testing to verify the proposed benefits of our system and to receive user feedback for further improvements. To begin to accomplish, this we have already deployed our system with our collaborators at Hokkaido University and Kansai University, who work on molecular robot research in wet laboratories.

Following initial user testing, we are also planning to expand the range of simulation models included within our CCE to enable more users to benefit from our system and to obtain more feedback for continuing to improve our platform. Part of expanding our user base will also include finalizing our mobile VR client application which will reduce the hardware requirements of users further.

## 5 VR viewer for microscope images

In parallel to the development of our CCE, members of our team are working on additional VR environments to be added to our CCE to promote further research collaboration in the CCE. One of these environments is a VR viewer for microscope images. Microscopes, such as atomic force microscopes and confocal microscopes, are essential in the rational design of molecular robot parts [3]. However, visualizing these images is often challenging because they are provided in a volumetric (three-dimensional) form.

We adopted a simple, effective visualization approach in our simulation. In confocal microscopy imaging [57], for a sequence of two-dimensional images, which are slices of a three-dimensional volume, the volume is rendered as a stack of transparent, double-sided planes in the simulation space. Our system also supports additional information, such as slice position and color, which reflect the Z position of the XY slice and its color, respectively.

Currently, the method supports volumes with up to 512 × 512 × 512 resolution. Figure 8 shows a volume composed of 296 × 512 × 512 slices (with different colors for each slice) rendered via our approach. VR visualization has great potential to improve the perspective of target molecular structures.

## 6 Application to molecular robotics

COVID-19 has dramatically changed the world. One specific area of change that has touched the lives of nearly everyone is the shift in drug and vaccine design [58]. Similar to how lipid nanoparticle-containing mRNA technologies have recently emerged and become the most common COVID-19 vaccine technology [59], nanorobots, which have sensors and actuators [60], are expected to be one of the next major leaps in drug development [61–63].

Molecular robots [64] could be used to build nanorobots, as well as mechanical nanorobots and molecular machines [65, 66]. A molecular robot is made of biomolecules, such as DNA and proteins, and the robots often consist of a container to separate their inner state from their environment, sensors to obtain information from their environment, a processor to handle the information, and actuators to move [67]. In previous work by Kassem et al. molecular machines that could selectively transport a substrate between different activation sites have been demonstrated as one application [63].

The objective of our VR simulation is to observe the dynamics of a wide range of molecular interactions including DNA nanostructures and microtubule motion pattern formation to find a good initial distribution of atomic molecular models to which conventional molecular simulation can be applied. By simulating at atomic levels such as DNA nanostructures we aim to identify and understand the building blocks of molecular robots [48].

Then from there, we can add the findings of such work into higher-level coarse-grained simulations focusing on molecular robots [3]. Due to the wide range of molecular interactions that we are targeting, we strongly believe that visual inspection is one of the most promising ways to observe interesting molecular interaction processes which last to some extent on the way to a steady state or dispersed state [20]. VR is also useful to observe subtle differences in self-organized molecular structures such as hydrogen bond distance and directions, hydration of water molecules on bio-molecular surfaces, etc. [3, 48].

A molecular robot approach is superior to a mechanical nanorobot approach in terms of miniaturization because a molecular robot can use sources of chemical energy, such as adenosine triphosphate (ATP) [66], which is abundant in the human body. This approach is also superior to a molecular machine approach with respect to part types; molecular robots can use DNA strands of any length and any kind of proteins as molecular parts [56, 68]. The main challenge for molecular robots is to assemble the molecular parts into a system, similar to living organisms [64, 67].

Several molecular robot prototypes have been reported, including self-propelled liposomes [38–40], muscle-like actuators [41, 42], and swarm molecular robots [43, 44] (Table 1). Thus, the concept of molecular robots is feasible and promising, although there is still great scope for improvement and development.

Although much fundamental research has been reported in the field, practical applications of molecular robotics remain in the preliminary stages. To solve this problem, we have been developing a cloud-based CCE for rational molecular robot part design using VR [3, 4] and real-time AI [51].

### 6.1 Current limitations

Our own collaborators and other teams around the world have found that a critical issue is the acceleration of the molecular part design cycle [38–44]. Molecular robots often use DNA nanostructures and chemically modified proteins as molecular parts [44]. The great advantage of using DNA nanostructures is the availability of computer-aided design (CAD) systems that can automatically generate the sequence information for DNA strands for a desired nanostructure shape [68]. However, because there are gaps between DNA sequence-based design and the actual DNA nanostructure observed by atomic force microscopy, iterative design is often needed [56]. Simulation is one of the technologies that may be able to shorten the iterative design process though.

For simulation technologies, there is also a lack of rational molecular design tools applicable to molecular robot parts, such as DNA origami, protein complexes, or microtubules. Consequently, conventional molecular dynamics simulations have been used, but these often take more than several weeks to run, even on high-performance computers [48]. This has restricted collaboration further, limiting the number of perspectives and ideas available during the development cycle according to our collaborators in wet laboratories [42–44].

In an effort to reduce some of the simulation challenges, previously we created a coarse-grained model utilizing GPGPU to accelerate the simulation of microtubule gliding assays [20], which are a common wet experiment used for microtubule dynamics research that is applied to molecular robotics design. This model was successfully applied to the study of microtubule motion patterns by Inoue et al. [43]; however, it only ran locally and required expensive hardware which limited its use, this motivated us to seek another way to simulate to enhance the usability of our models.

### 6.2 Proposed work

To accelerate the molecular part design cycle, we have begun to develop a cloud-based virtual reality co-creation environment (CCE) augmented by real-time artificial intelligence (AI). The CCE aims to provide molecular objects and equipment in virtual reality as a service via the cloud so that researchers can collaborate remotely. To compensate for the latency caused by the distance between researchers, real-time AI technology, known as real-time inference and training (RTIT), has been implemented and plays an essential role in ensuring remote operation feels natural in the cloud-based virtual reality (VR) environment [3, 4, 51].

## 7 Ethical considerations

A CCE is similar to conventional CAD software, in that it could be used for designing beneficial or harmful things. Although this problem is difficult to address, CAD software has been accepted despite the fact that it can be used to design anything.

The democratization of knowledge is another challenging question. It would likely be beneficial to educate more people with the aim of benefitting society as more people could then participate in industries that aim to solve future medical problems or create innovative materials. However, some people may have bad intentions, and limiting access based on intention is a challenging problem.

The biological results that may be produced after the CCE is used for the design stage would need their own ethical consideration, which lies beyond the scope of this paper on the CCE. We are currently talking with ethics researchers about the innovative technologies in this project, such as molecular robotics [69].

## 8 Conclusions

By following the design philosophy described in Section 3 and using the technologies discussed in Sections 4–6, we intend to create a cloud-based molecular VR CCE that is widely available and enhances the abilities of molecular biologists as they work towards developing molecular robots. In addition, as we solve the many research challenges in our own work, each future solution, which will be explored in future papers, should have applications outside of our own work.
